# The Effect of Self-Selected Exercise Workloads on Perceived Enjoyment and Self-Efficacy in Sedentary Adults

**DOI:** 10.3390/bs12070224

**Published:** 2022-07-10

**Authors:** Peyton Waaso, Natalie Gofton, Micah Zuhl

**Affiliations:** School of Health Sciences, Central Michigan University, Mount Pleasant, MI 48706, USA; waaso1pr@cmich.edu (P.W.); gofto1nm@cmich.edu (N.G.)

**Keywords:** perceived enjoyment, self-efficacy, self-selected, interval, continuous

## Abstract

Self-selected workloads are shown to be more enjoyable than researcher-selected workloads. In addition, it is unclear if sedentary adults find aerobic interval or continuous exercise more pleasant. Therefore, the primary purpose of this study was to determine the effects of two acute bouts (interval vs. continuous) of self-selected moderate-intensity treadmill exercises on perceived enjoyment and self-efficacy towards exercise in a sedentary cohort. **Methods:** Sixteen sedentary adults completed two 30 min bouts of moderate-intensity treadmill activity, one interval and one continuous. Participants blindly (could not see speed, grade, and heart rate) selected their own treadmill workload with guidance from the Borg RPE 6–20 scale. Post-exercise self-efficacy and perceived enjoyment were assessed using the Self-Efficacy for Exercise Scale and the Physical Activity Enjoyment Scale, respectively. Exercise workloads using treadmill speed and grade and exercise heart rate were compared between trials. **Results:** No significant differences were found between conditions for self-selected workloads (*p* = 0.62), self-efficacy (*p* = 0.58), perceived enjoyment (*p* = 0.41), and heart rate (*p* = 0.12). **Discussion:** Sedentary individuals reported no difference in self-efficacy or perceived exercise enjoyment. Participants were, however, adequate in self-selecting their own intensities with RPE guidance as there were no differences in the workloads across conditions. These results suggest that when able to self-select moderate-intensity exercise workloads, sedentary individuals equally enjoy both interval and continuous exercise.

## 1. Introduction

The number of sedentary individuals in the United States and globally has expanded over time, and this inactivity is associated with an increased risk of cardiovascular and metabolic diseases; a 20–30% increased risk for all-cause mortality, in comparison to those who are physically active [1,2]. Despite the known benefits of physical activity (PA), data suggest that nearly 50% of adults taking part in an exercise training program drop out within the first 6 months [3]. The authors cited lack of motivation, access to equipment, and self-efficacy as some of the reasons for the recidivism. In addition, beginning and adhering to an exercise training program often require both intrinsic and extrinsic factors, such as confidence, time, money, physical appearance, and enjoyment of the exercise.

Perceived enjoyment of exercise along with self-efficacy, or confidence in performing a particular type of exercise, are strong predictors of adherence. For example, exercise enjoyment was the strongest predictor of weekly participation in an 8-week exercise program. The intervention promoted enjoyment through participant input and decision making about exercise [4]. Moreover, physical activity enjoyment and self-worth were central factors in explaining long-term exercise adherence among women [5]. The authors recommended that increasing relative enjoyment and self-regulation to support confidence would improve exercise adherence [5]. In addition, designing exercise programs that meet global fitness and health trends has been recommended to improve physical activity [6]. These findings indicate that identifying enjoyable exercises and encouraging exercise autonomy (and confidence) may improve adherence.

Studies have shown interval aerobic training to be a more enjoyable or as enjoyable form of exercise compared to continuous aerobic training. Positive psychological outcomes have been reported among sedentary obese women after participation in interval exercise; however, many of these studies employed moderate-intensity continuous training (MICT) in comparison to HIIT, with minimal attention paid to moderate-intensity interval training (MIIT) [7,8,9,10,11]. MICT typically involves continuous aerobic workloads of 40–60% VO_2_Reserve or heart rate reserve, a 12–13 on the Borg rating of perceived exertion (RPE) scale (scaled 6–20), or an equivalent of 3–5.9 METs [12]. MIIT requires similar workloads to MICT, but is broken up into interval bouts, similar to HIIT. A study by Coswig et al. demonstrated that MIIT is superior to MICT for improvements in blood pressure, the 6 min walk test, and reduced detraining effects [13]. In sedentary populations, this could be an effective means of exercising as the training intensities are similar despite low activity profiles, thereby having the potential to increase perceived enjoyment, self-efficacy, and adherence rates. Conversely, HIIT running is associated with prolonged recovery and could result in longer durations between exercise days, potentially increasing dropout rates [13,14]. MIIT could thus impact enjoyment and exercise confidence in a sedentary group by reducing recovery time and negative feelings towards exercise [15].

Adherence rates and enjoyment in an exercise training program can be connected to the idea behind self-selection (autonomy) of exercise intensity based on the affect system. This connection, according to Cacioppo and Bernston, relates to the individual desire to partake in activities that offer positive or appetitive information processing and to avoid situations that offer aversive or negative information processing [16]. High-intensity exercise, for example, could be perceived as offering aversive health effects and possessing the potential for danger, meaning people may be less likely to participate in that type of activity. Self-selected or “preferred” exercise intensities have been shown to trend towards prescription ranges and produce effects similar to pre-determined workloads [17]. In the review by Ekkekakis, individuals in a variety of exercise training studies have been found to be able to adequately judge and perform higher intensities when offered the ability to self-select a particular intensity [17]. Recreational exercisers typically self-select their own intensities; therefore, determining which self-selected intensities produce greater feelings of enjoyment and confidence could indicate which exercises are most appropriate to suggest for individuals who find it difficult to adhere to exercise.

The purpose of this study is to determine the effects of two acute bouts of self-selected moderate-intensity treadmill trainings (interval vs. continuous) on perceived enjoyment and self-efficacy towards exercise in a sedentary population.

## 2. Materials and Methods

### 2.1. Participants

Sixteen sedentary adults between the ages of 23 and 54 were recruited to participate in a three-day study. Individuals were considered sedentary if they did not adhere to the ACSM’s current exerciser classification, which includes performing planned, structured PA of at least 30 min at a moderate intensity for 3 or more days per week over the past 3 months [12]. Individuals were excluded if they had been diagnosed with cardiovascular, metabolic, renal, pulmonary, bone, or neuromuscular diseases; if they experienced chest pain during exertion; were currently pregnant; were currently COVID-19 positive or had tested positive for COVID-19 within the previous 90 days. All participants provided written informed consent for inclusion prior to participating in the study. The study was conducted in accordance with the Declaration of Helsinki, and the protocol was approved by the Ethics Committee of Central Michigan University (protocol 2021-519).

### 2.2. Study Design

A single-blind cross-over study design was implemented for this study. On day one, anthropometric measurements were evaluated using a calibrated in-lab stadiometer and scale (Seca 213; Hamburg, Germany). Participants were then familiarized with the study treadmill operations and were educated on the use of the Borg RPE scale. Next, they were asked to complete an 8 min walking sub-maximal exercise test to estimate aerobic fitness levels [18,19]. Participants were then randomized to either perform 30 min of self-selected MIIT or MICT first. A minimum 3-day washout period was implemented, and participants completed both conditions at similar times of day and refrained from alcohol and caffeine intake at least 24 h prior to participation.

### 2.3. Exercise Interventions

The target intensity for the 30 min trials was an RPE value of 13 (“somewhat hard”). Participants self-selected their treadmill speed and grade (i.e., workload) to match the targeted RPE value. However, they were blinded to the treadmill values (could not see speed and grade). Treadmill workloads (Cardiac Science TM55; Bothell, WA, USA) were adjusted by the participant using an attached keyboard to the treadmill rail, and treadmill workloads were displayed on a computer rotated away from the participant. Participants were allowed to adjust treadmill speed and grade at any point during exercise. In addition, heart rate was monitored throughout the exercise using a chest strap and watch (Polar FT1, Kempele, Finland). Participants were also blinded from knowing their exercising heart rate. Participants completed both protocols on the same treadmill and were assessed by the same primary investigator across conditions.

#### 2.3.1. MIIT

Each participant completed a short mood questionnaire prior and immediately after exercise (see Section 2.4 below). The MIIT session began with a 5 min continuous walking warm-up at a treadmill workload of 3.2 kmh (2.0 mph), 0% grade. Seven intervals were then completed, consisting of a one-minute bout at a self-selected RPE of 15 (hard) and a two-minute active recovery at an RPE of 11 (light). The participant self-selected the treadmill speed and grade using the keyboard controller to achieve an RPE of 15 during the interval bout and during the recovery bout. Treadmill workloads and heart rate were recorded each minute. Exercise was concluded with a 5 min walking cooldown at 3.2 kmh (2.0 mph). The RPE goal of the total session was 13, which included a target of 15 for the interval and 11 for recovery for an average of 13.

#### 2.3.2. MICT

The MICT protocol consisted of the same warm-up and cooldown as the MIIT protocol, along with the pre-exercise mood assessment. After the warm-up period, each participant self-selected a speed and grade consistent with an RPE of 13 (somewhat hard) for 20 min. If RPE were to reach a level above or below a 13 throughout the protocol, participants were encouraged to adjust the treadmill workloads (either speed or grade, or both). Treadmill workload and exercise heart rate were recorded every minute.

#### 2.3.3. Exercise Calculations

Treadmill workload was calculated following the ACSMs metabolic equations for gross VO_2_, which is dependent upon treadmill speed and grade. Two equations were used depending on walking and running speeds. The walking equation was used for speeds of 3.06 to 5.96 km per hour, kph (1.9–3.7 miles per hour, mph). Using a speed (S) conversion factor of 26.82 to convert mph to meters per minute, and G representing grade in percent. Note, 3.5 mL·kg^−1^·min^−1^ represents resting VO_2_. The walking equation reads:

Walking VO_2_ (mL·kg^−1^·min^−1^) = (S × 0.1) + (S × G × 1.8) + 3.5.

For speeds greater than 5.96 kpm (3.7 mph), the running equation was used, utilizing the same speed and grade components as the walking equation. The running equation reads:

Running VO_2_ (mL·kg^−1^·min^−1^) = (S × 0.2) + (S × G × 0.9) + 3.5.

The running VO_2_ metabolic equation is most accurate for speeds greater than 8 kph (5 mph), but if an individual was exercising on the treadmill at a speed >5.96 kph (3.7 mph), for example, then the running equation was used [12].

### 2.4. Questionnaires

A mood questionnaire was administered before and after the MIIT and MICT sessions. Using a 10-point scale (0—not at all; 10—very), participants were asked to assess their state of anxiousness, liveliness, sadness, hopelessness, cheerfulness, annoyance, anger, happiness, and uneasiness. The scale was designed to capture acute changes in various subjective feels related to mood after both MIIT and MICT exercise.

At the cessation of each treadmill protocol, two scales were administered, including the Physical Activity Enjoyment Scale (PACES) and the Self-Efficacy for Exercise (SEE) scale [20,21]. The SEE scale was used to assess self-efficacy towards exercise and was the first scale administered after the cooldown period. This is a 10-point Likert scale with 9 items that can score from 0–90, with higher scores indicating higher self-efficacy or confidence towards exercise. Participants were asked to indicate feelings of confidence associated with different situations or events involved in exercise. Scores were calculated by totaling responses to each question. According to Resnick & Jenkins, this scale has shown high internal consistency (0.92) and predicts exercise activity well [21]. The PACES scale was used to assess perceived enjoyment of exercise and was the second scale administered after the cooldown period following the acute mood states scale. This is a 7-point Likert scale with 18 items that can score from 7 to 126, with higher scores representing more positive feelings associated with exercise. Participants were asked to indicate how strongly they felt between two extremes for each item. Scores were calculated by totaling each item, noting reversal of scoring when indicated by the scale.

### 2.5. Statistical Analysis

All data are presented as mean ± standard deviation unless otherwise detailed. A priori calculation was used to determine sample size with power of 0.80 and an alpha level of 0.05 (G*Power, Dusseldorf, Germany). The criteria for selected studies were acute exercise (continuous vs. interval), measures of physical activity enjoyment (using PACES), and mood. The expected effect size for PACES was 1.0 [7], which estimates a minimum sample size of 10 to detect statistical significance. Student’s *t*-test analysis was conducted to compare average heart rate, average treadmill workload, and results of the PACES and SEE surveys between MICT and MIIT. In addition, the acute changes (pre vs. post) in mood for each variable (anxiousness, liveliness, sadness, hopelessness, cheerfulness, annoyance, anger, happiness, and uneasiness) were calculated, and change was compared between MICT and MIIT using a two-way ANOVA analysis with independent variables being exercise trial (MICT and MIIT) and time (pre- and post-exercise). Mean differences (post- minus pre-exercise) between both trials (MICT vs. MIIT) were also compared to account for baseline levels of mood states using a paired *t*-test. Pearson correlation analysis was used to evaluate the relationship between PACES and SEE scores for all data combined (MICT + MITT). Data were analyzed using the Statistical Package for Social Sciences software program (SPSS, version 18.2.1; Chicago, IL, USA). A *p*-value of <0.05 indicates statistical significance.

## 3. Results

Demographic characteristics of the study participants are displayed in Table 1. Outcomes are reported in Table 2. No significant differences were found between MICT and MIIT conditions for self-selected mean treadmill workloads (18.49 ± 5.50 vs. 18.95 ± 4.78 mL·kg^−1^·min^−1^, respectively, *p* = 0.62). Similarly, no difference was detected between MICT and MIIT trials for average heart rate (125 ± 17 vs. 132 ± 18 bpm, respectively, *p* = 0.12).

**Figure 1:**
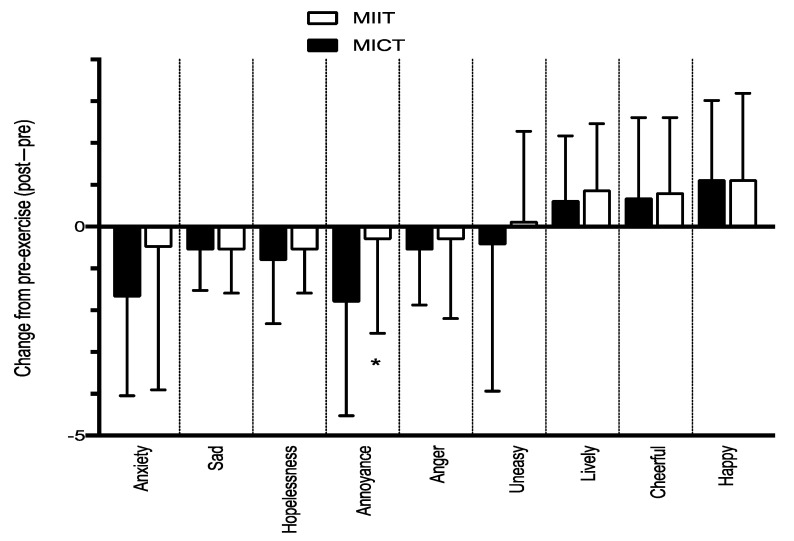
Acute mood changes after MICT and MIIT trials. * Significant difference in changes in self-reported annoyance after MICT vs. MIIT, *p* < 0.01.

There were also no differences found between MICT and MIIT for the PACES (98.43 ± 17.84 vs. 96.56 ± 19.81, respectively, *p* = 0.41, see Table 2) and SEE (41.38 ± 23.29 vs. 43.06 ± 22.20, respectively, *p* = 0.58). The between trial differences in acute mood changes are presented in Figure 1. The two-way ANOVA for differences in self-reported annoyance revealed a significant effect of time (pre vs. post), F (1, 30) = 5.83, *p* = 0.02. The pre to post change was significant within the MICT trial (2.98 ± 3.13 vs. 1.25 ± 2.06, *p* = 0.01), but not the MIIT trial. Similarly, the differences in self-reported anxiety revealed a significant effect of time (pre vs. post), F (1, 30) = 12.87, *p* = 0.01. The pre to post change was significant within the MICT trial (3.31 ± 2.70 vs. 1.31 ± 1.53, *p* = 0.004), but not the MIIT trial. No other acute differences were detected. To account for baseline levels of mood, the mean difference (e.g., post minus pre) between trials was compared. Only self-reported annoyance was significantly different between MICT and MIIT trials (−1.81 ± 2.73 vs. −0.31 ± 2.24, *p* = 0.007, respectively). Figure 1 shows the mean differences for each of the self-reported acute mood changes.

**Figure 2:**
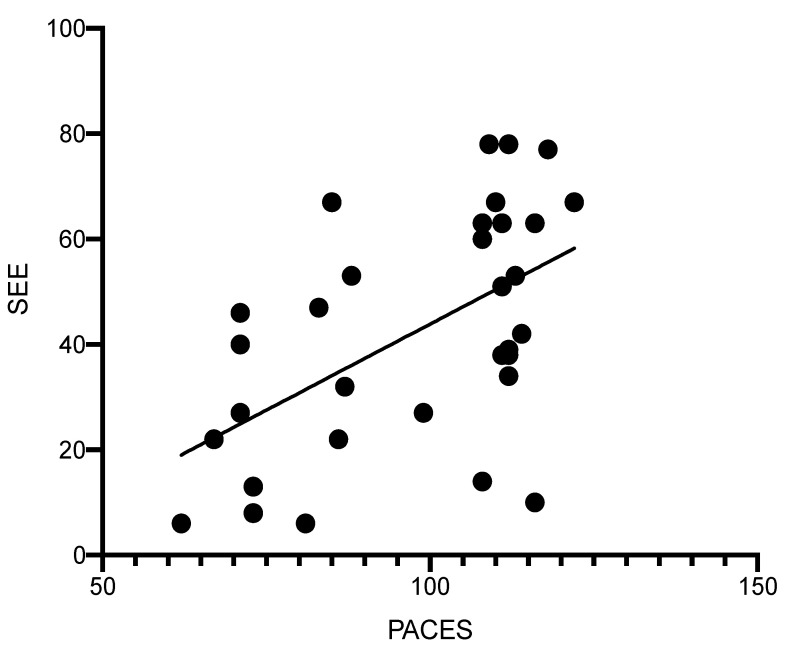
Relationship between SEE and PACE scores for both trials combined (MIIT + MICT). • represents each participant. (R^2^ = 0.29, *p* = 0.001).

The relationship between PACE and SEE scores from both MICT and MIIT was compared using the Pearson correlation analysis. A significant, but small relationship was detected for all exercise sessions combined (R^2^ = 0.29, *p* = 0.001, Figure 2).

## 4. Discussion

The results suggest that sedentary individuals do not perceive differences in enjoyment or feel more competent in their ability when performing either MICT or MIIT as both outcomes (PACES and SEE) were not different between trials. This may be explained by the self-selection component of the intervention, and by the targeted intensity. Specifically, individuals blindly selected the same treadmill workloads and achieved similar heart rate values during both trials, which would evoke a comparable stress response and corresponding feelings of enjoyment and self-efficacy. In addition, the moderate-intensity exercise is more logical and aligns with beginning exercise intensity recommendations for sedentary adults [22].

As mentioned, the results of the PACES and SEE questionnaires were not different between the MICT and MIIT trials. The PACES questionnaire has been frequently applied to assess enjoyment of physical activity with higher scores reflective of greater enjoyment [20]. Thum et al. (2017) reported significantly higher PACE scoring from 12 recreationally active men and women after high-intensity interval training (HIIT) compared to MICT [23]. The recruitment of active participants versus sedentary may explain the conflicting results. In a follow-up study, the same research group reported no difference in PACES scores among sedentary individuals who completed various HIIT protocols compared to MICT [24]. Soylu et al. (2021) reported higher exercise enjoyment among recreational active individuals after a self-selected HIIT intervention versus MICT [25]. This study most closely aligns with the current study due to the self-selection nature of the exercise protocols. Participants completed either self-selected running intervals or continuous jogging around a 400 m outdoor athletics track. Again, the explanation could be the recruitment of active participants, but another reason may be that it was a training study. Exercise enjoyment has been reported to be lower after acute or single session high-intensity interval exercise bouts compared to continuous among sedentary adults [26]. Acute high-intensity interval exercise may initially be uncomfortable and result in feelings of dissatisfaction; however, as one continues to perform interval training, the improvement in physical fitness may begin to influence feelings of enjoyment. This is evident by the increase in PACES scores across a 12-week HIIT program compared to MICT among overweight young women [27]. The null findings in the current study may be due to both the implementation of moderate-intensity interval exercise and allowing sedentary adults to self-select their own exercise intensity (under guidance of RPE). It is important to highlight that the PACES scores reported (98.43 ± 17 and 96.56 ± 19.81 for MIIT and MICT, respectively) align with similarly high values reported in exercise programs. For example, a ballroom dancing intervention resulted in PACES scores of 108 among healthy Filipino Americans [28]. Moreover, PACES scores ranged from 85–87 for various acute HIIT sessions among sedentary individuals, and show that perceived enjoyment appeared to be higher in the current study [24].

Evidence suggests that those with stronger self-efficacy or perceptions of their own ability to successfully perform an exercise activity are more likely to adhere to a given exercise program [29]. Limited data exist examining self-efficacy comparisons between interval and continuous exercise regimes. Previous work has demonstrated lower self-efficacy among sedentary middle-age adults towards acute HIIT and vigorous continuous exercise compared to moderate-intensity continuous exercise [30]. Conversely, no difference in self-efficacy was reported among individuals with type-2 diabetes mellitus who participated in either a HIIT or MICT program; however, comparisons were made at the end of a 12-week intervention [31]. The instrument used in the current study was the 0–90-point Self-Efficacy for Exercise (SEE) scale with higher scores interpreted as greater self-efficacy. The scores reported were not different between the MICT and MIIT trials; however, values were below the half-way point of scale, which may indicate moderate to low levels of self-efficacy. One explanation may be the unfamiliarity with the operation of the treadmill during each exercise session. A familiarity trial was implemented, but the lack of competency may have remained.

Data from both trials were combined to evaluate the relationship between exercise enjoyment (PACES) and exercise self-efficacy (SEE), and a weak, but significant correlation was observed. This indicates that higher PACES align with high SEE scores during either form of self-selected moderate-intensity exercise. This relationship is not causal (one outcome predicting the other); however, Hu et al. reported that exercise self-efficacy levels predict physical activity enjoyment, and therefore, one’s belief in their ability to exercise may be the key to identifying enjoyable activities [32].

Acute changes in self-reported anxiety and annoyance were lower after the MICT trial (pre vs. post), and the mean difference in annoyance was lower in the MICT compared to the MIIT trial. The data do not suggest that acute mood changes influenced either exercise enjoyment or self-efficacy; however, evidence has demonstrated that high levels of annoyance and anxiety negatively impact adherence to exercise programs [33,34]. Moreover, acute exercise has been shown to significantly alter levels of anxiety among both healthy individuals and those suffering from both physical and mental disorders [35]. Acute changes in anxiety have also been reported to be lower after a bout of self-selected exercise compared to prescribed [36]. Therefore, the changes in mood may be explained by allowing individuals to self-select their own exercise workloads.

Self-selected treadmill workloads were similar across both trials with individuals selecting 18.49 and 18.95 mL·kg^−1^·min^−1^ for MICT and MIIT, respectively. These values are slightly over 5 METs and fall in line with current moderate-intensity guidelines [12]. Target intensity was based on an RPE value of 13 out of 20, or “somewhat hard”, and treadmill workloads were adjusted by the participant based on how they perceived the work. They were also blinded to the actual speed and grade of the treadmill throughout each trial to prevent subjective bias. These findings highlight the ability of sedentary individuals to self-guide exercise intensity when given RPE instruction. The reliance on RPE for selecting exercise intensity has grown based on the ease of implementation and ecological usefulness [37]. In addition, RPE use during exercise training helps to promote exercise adaptation while minimizing overtraining injuries by supporting one’s self-awareness and perception of their own physical abilities [38]. Moreover, the opportunity to be in control of ones exercising experience has previously been shown to produce greater feelings of interest, autonomy, and perceived enjoyment, and may also help to explain high perceived enjoyment scores in the study [39]. A review of studies examining self-selected exercise showed that sedentary obese participants likely self-select exercise intensity based on pleasure [17]. Future studies should compare these type of subjective feelings as well as dropout rates between self-selected and researcher-controlled exercise using a moderate-intensity interval protocol.

Similarly, there were no significant differences in heart rate responses between trials, which is not surprising due to the self-selection of similar exercise workloads in both trials. The choosing of similar MET values would elicit a similar cardiovascular and hemodynamic response. Mean exercise heart rates were 125 and 132 bpm in MICT and MIIT, respectively. The original design of the Borg RPE scale was based on an estimated exercising heart rate. In practice, a target RPE of 13 (which was the goal in the current study) would align with a heart rate of 130 bpm [40,41]. The close similarity in heart rate between trials is surprising and demonstrates that participants were able to successfully utilize the Borg RPE scale after explanation from the research team during the initial testing session. We were unable to locate similar studies that compared heart rate responses to acute self-selected MICT and MIIT protocols. In studies that compare HIIT to MICT, heart rate responses are far more variable as HIIT prescriptions typically involve intensities during exercising intervals of 90–95% HRmax and recovery periods of 60–70% HRmax compared to MICT, which generally involves exercise intensities of 70% HRmax throughout [42]. Using moderate-intensity exercise for both protocols eliminated those potential differences in heart rate.

This study is not without limitations. Notably, the study’s small sample size. Participants were recommended not to change their daily activities, but food logs and physical activity logs could have aided in more control within the current study. The PACES and SEE scales used in this study have not been validated among diverse populations. In a study assessing the PACES validity in older sedentary adults, 91% of sampled participants (out of 179) were Caucasian [43]. In another study assessing the reliability and validity of the SEE scale, 98% (out of 187) were Caucasian [21]. Because of these previous validation studies, it is unknown whether the scales are equally applicable to minority populations based on cultural and societal differences. The mood questionnaire administered has not been validated and was used to capture different feelings without requiring participants to complete numerous scales which may have innately altered the participant’s mood.

## 5. Conclusions

As physical activity levels decline, it is important to identify exercise regimes that are likely to support sustained adherence. Another important factor that may influence adherence is the consideration of current fitness trends, such as programming for weight loss, technology advances (wearables), outdoor activities, and interval forms of exercise [6]. This study demonstrated high levels of perceived enjoyment during both self-selected moderate-intensity exercise sessions (MICT vs. MIIT), but no difference between trials among sedentary adults. Further, this study highlights the ability of sedentary individuals to self-select appropriate intensities while utilizing the Borg RPE scale. In summary, the type of moderate-intensity exercise (interval vs. continuous) may not matter when sedentary individuals are able to self-select exercise workloads under the guidance of RPE, and demonstrates the possible role of exercise autonomy. Future research should focus on the impact of self-selected and researcher-selected workloads utilizing a moderate-intensity interval protocol to understand the mechanisms behind workload selection, self-efficacy, perceived enjoyment, and their impacts on adherence rates.

## Figures and Tables

**Table 1:** Participant Demographics.

	Female (*N* = 10)	Male (*N* = 6)
Age (years)	33.4 ± 10.01	30.33 ± 6.58
BMI (kg/m^2^)	24.06 ± 3.94	25.32 ± 4.01
Estimated VO_2_max (mL·kg^−1^·min^−1^)	29.66 ± 7.48	39.38 ± 8.26

Abbreviations: BMI—body mass index; VO_2_max—maximal oxygen consumption.

**Table 2:** Trial Outcome Data.

	MICT	MIIT	*p*-Value
Workload (mL·kg^−1^·min^−1^)	18.49 ± 5.50	18.95 ± 4.78	0.62
Heart Rate (bpm)	125 ± 17	132 ± 18	0.12
PACES	98.43 ± 17	96.56 ± 19.81	0.41
SEE	41.38 ± 23.29	43.06 ± 22.20	0.58

Abbreviations: MICT—moderate-intensity continuous training; MIIT—moderate-intensity interval training; bpm—beats per minute; PACES—physical activity enjoyment scale; SEE—self-efficacy for exercise scale.

## Data Availability

The data presented in this study are available on request from the corresponding author. The data are not publicly available due to informed consent compliance.
